# Genome‐wide association analysis reveals distinct genetic architectures for single and combined stress responses in *Arabidopsis thaliana*

**DOI:** 10.1111/nph.14165

**Published:** 2016-09-08

**Authors:** Nelson H. Davila Olivas, Willem Kruijer, Gerrit Gort, Cris L. Wijnen, Joop J. A. van Loon, Marcel Dicke

**Affiliations:** ^1^ Laboratory of EntomologyWageningen UniversityPO Box 166700 AAWageningenthe Netherlands; ^2^ BiometrisWageningen UniversityPO Box 166700 AAWageningenthe Netherlands

**Keywords:** abiotic stress, biotic stress, combined stresses, genome‐wide association, specialist herbivores

## Abstract

- Plants are commonly exposed to abiotic and biotic stresses.
- We used 350 *Arabidopsis thaliana* accessions grown under controlled conditions. We employed genome‐wide association analysis to investigate the genetic architecture and underlying loci involved in genetic variation in resistance to: two specialist insect herbivores, *Pieris rapae* and *Plutella xylostella*; and combinations of stresses, i.e. drought followed by *P. rapae* and infection by the fungal pathogen *Botrytis cinerea* followed by infestation by *P. rapae*.
- We found that genetic variation in resistance to combined stresses by drought plus *P. rapae* was limited compared with *B. cinerea* plus *P. rapae* or *P. rapae* alone. Resistance to the two caterpillars is controlled by different genetic components. There is limited overlap in the quantitative trait loci (QTLs) underlying resistance to combined stresses by drought plus *P. rapae* or *B. cinerea* plus *P. rapae* and *P. rapae* alone. Finally, several candidate genes involved in the biosynthesis of aliphatic glucosinolates and proteinase inhibitors were identified to be involved in resistance to *P. rapae* and *P. xylostella*, respectively.
- This study underlines the importance of investigating plant responses to combinations of stresses. The value of this approach for breeding plants for resistance to combinatorial stresses is discussed.

## Introduction

During their life cycle, plants are exposed to diverse abiotic stresses, such as drought, flooding, heat, cold, nutrient deficiency or ozone (Shinozaki & Yamaguchi‐Shinozaki, 2007; Roy *et al*., 2011; Fahad *et al*., 2015; Mickelbart *et al*., 2015), and biotic stresses, such as attack by bacteria, fungi, viruses, insects or parasitic plants (Howe & Jander, 2008; Mithofer & Boland, 2012; Pieterse *et al*., 2012). Substantial progress has been made in the identification of genes that provide resistance to individual stresses (Smith & Clement, 2012). However, in natural ecosystems, plants suffer from combinations of stresses that occur simultaneously or sequentially. Recent studies have addressed this by investigating the phenotypic effect, transcriptomic changes and genetics underlying responses to combined stresses (De Vos *et al*., 2006; Atkinson *et al*., 2013; Rasmussen *et al*., 2013; Kissoudis *et al*., 2014). These studies have concluded that the effect of a combination of stresses can often not be predicted from the single stress effect at the phenotypic, transcriptomic or genetic level.

Herbivory by insects is one of the major stresses faced by plants: one‐quarter of all known eukaryotic species are insect herbivores (Futuyma & Agrawal, 2009). As a result of the strong selection pressure imposed on plants by insects, plants have evolved mechanisms to protect them from insects (Kessler & Baldwin, 2002; Schoonhoven *et al*., 2005). Plant traits that influence the degree of damage caused by insects can be classified into resistance (traits that limit the damage by the insect) and tolerance (traits that allow plants to compensate for insect damage) (Strauss & Agrawal, 1999; Stout, 2013). Furthermore, resistance and tolerance are mediated by distinct genetic mechanisms (Strauss & Agrawal, 1999; Carmona *et al*., 2011; Karinho‐Betancourt & Nunez‐Farfan, 2015). Resistance can be further divided into constitutive or induced defences (Schoonhoven *et al*., 2005; Mithofer & Boland, 2012; Stout, 2013). One of the best studied defence mechanisms of plants against insects is the myrosinase–glucosinolate system in the Brassicaceae family (Hopkins *et al*., 2009; Mithofer & Boland, 2012). Glucosinolates are hydrolysed by myrosinase enzymes on insect herbivory and their breakdown products are toxic to generalist insect herbivores (Fahey *et al*., 2001; Kliebenstein *et al*., 2005; Brachi *et al*., 2015). However, specialist insects, such as *P. rapae* and *P. xylostella*, have developed detoxification mechanisms and do not seem to be affected by the myrosinase–glucosinolate defence of brassicaceous plants (Wheat *et al*., 2007; De Vos *et al*., 2008; Müller *et al*., 2010). These two insect species are major pests in several crops from the *Brassica* genus (e.g. broccoli, cabbage, cauliflower) worldwide. For example, annual control costs of *P. xylostella* are estimated to be nearly US$ 4–5 billion (Zalucki *et al*., 2012). A good understanding of the genetic architecture of plant resistance against these insects and the identification of molecular mechanisms behind resistance would provide breeders with better tools to develop crops that are more resistant to these insect species.

In nature, insect herbivory commonly occurs simultaneously or sequentially with other abiotic and biotic stresses (Rizhsky *et al*., 2004; Mittler & Blumwald, 2010; Vile *et al*., 2012; Prasch & Sonnewald, 2013; Rasmussen *et al*., 2013; Kissoudis *et al*., 2014; Rivero *et al*., 2014; Sewelam *et al*., 2014; Stam *et al*., 2014; Suzuki *et al*., 2014). Several studies have shown that, when plants experience a certain stress, this may compromise the plant's ability to respond to subsequent stresses (Suzuki *et al*., 2014; Ramegowda & Senthil‐Kumar, 2015). Therefore, the study of single stresses does not provide good predictors of plant responses to multiple stresses. Plant hormones have emerged as major players in the control of the signal transduction pathways that regulate stress responses (Erb *et al*., 2012; Pieterse *et al*., 2012). Jasmonic acid (JA), salicylic acid (SA) and ethylene (ET) have emerged as important signalling molecules in plant defences against pathogens and insects, whereas abscisic acid (ABA) is important for resistance to abiotic stresses (Shinozaki & Yamaguchi‐Shinozaki, 2007; Pieterse *et al*., 2012). JA activates signalling pathways that mediate responses against chewing herbivores and necrotrophic fungi, whereas SA underlies responses to biotrophic pathogens (De Vos *et al*., 2005; Pieterse *et al*., 2012). Phytohormonal signalling pathways interact through ‘crosstalk’, which may allow plants to respond in a fast and cost‐effective manner to stresses (Vos *et al*., 2013). Therefore, a good understanding of phytohormonal networks is needed to understand how plants tailor their response to different stresses. Hence, the analysis of responses to combinatorial stresses may yield information on the signalling nodes that are involved in the tailoring of the plant's adaptive response to stress combinations. To this end, we decided to study combinations of stresses to which individual responses are highly divergent, but, at the same time, regulated by interacting plant hormones. Therefore, we studied the interaction between *A. thaliana* and the specialist insect *P. rapae*, which is a JA inducer, the necrotrophic fungus *Botrytis cinerea*, which is a JA and ET inducer, and drought, which is an ABA and JA inducer (Verhage *et al*., 2010; Pieterse *et al*., 2012; Vos *et al*., 2013).

The model plant of genetic studies, *Arabidopsis thaliana*, displays natural genetic variation in developmental and physiological traits, as well as in resistance to biotic and abiotic stresses (McKay *et al*., 2003; Alonso‐Blanco *et al*., 2009; Baxter *et al*., 2010; Juenger, 2013; Easlon *et al*., 2014). In addition, natural genetic variation for resistance to specialist and generalist insects has been reported (Jander *et al*., 2001; Kliebenstein *et al*., 2002; Pfalz *et al*., 2007). The causal genes for variation in resistance against generalist insects have been successfully identified (mostly glucosinolate biosynthesis‐related genes) (Kliebenstein *et al*., 2002; Zhang *et al*., 2006). Less information is available on genes underlying variation in resistance to specialist insects and combined stresses (Kliebenstein *et al*., 2002; Pfalz *et al*., 2007; Kliebenstein, 2014). Quantitative trait locus (QTL) mapping using bi‐parental or multi‐parental populations has been traditionally employed for the identification of genes responsible for natural genetic variation for a trait of interest (Alonso‐Blanco & Koornneef, 2000; Koornneef *et al*., 2004). However, QTL mapping has a low resolution and requires a lot of time and resources (Doerge, 2002; Koornneef *et al*., 2004; Kloth *et al*., 2012; Weigel, 2012). In recent years, large collections of *A. thaliana* natural accessions have been genotyped and re‐sequenced, enabling genome‐wide association (GWA) studies in this model plant (Atwell *et al*., 2010; Weigel, 2012). GWA makes use of linkage disequilibrium (LD), when two loci in the genome are statistically more or less often inherited together as a result of recombination history, to associate genotypes with phenotypes. GWA overcomes several of the drawbacks of QTL mapping: GWA offers higher resolution (in some cases, down to the causal gene), is less time consuming and requires fewer resources, and considers more allelic diversity (Nordborg & Weigel, 2008; Zhu *et al*., 2008; Korte & Farlow, 2013). However, association mapping has some limitations: it requires large population sizes; it can generate a large number of false positives as a result of population structure; it has low statistical power to identify rare alleles; and it has difficulties in dissecting complex traits (many rare variants of large effects or many common variants of small effects) (Nordborg & Weigel, 2008; Zhu *et al*., 2008; Korte *et al*., 2012). Therefore, both strategies complement each other, leading to a higher power of finding causal genetic variation (Zhu *et al*., 2008; Myles *et al*., 2009; Brachi *et al*., 2010; Kloth *et al*., 2012).

Here, we used a collection of 350 *A. thaliana* accessions to explore the natural variation to a range of combinations of abiotic and biotic stresses. We chose the following stresses: drought, herbivory by caterpillars of *Pieris rapae* and *Plutella xylostella*, and infection by the necrotrophic fungal pathogen *Botrytis cinerea*. Under controlled conditions, we investigated the natural genetic variation in: resistance to two specialist insects, i.e. *P. rapae* and *P. xylostella*; resistance to combined stresses imposed by drought plus *P. rapae* and the plant pathogen *Botrytis cinerea* plus *P. rapae*. Resistance was quantified as the reduction in plant biomass under stress compared with non‐stress conditions. Furthermore, we used GWA mapping to gain insights into the genetic architecture of these traits and to identify regions in the genome associated with variation in resistance.

## Materials and Methods

### *Arabidopsis thaliana* Hapmap population

We used a collection of 350 *A. thaliana* accessions from the Hapmap population (http://bergelson.uchicago.edu/wp-content/uploads/2015/04/Justins-360-lines.xls). This population was developed from a global collection of 5810 accessions with the purpose to minimize redundancy and relatedness, a common problem in GWA studies (Atwell *et al*., 2010; Platt *et al*., 2010; Chao *et al*., 2012). This population has been genotyped for 248 584 bi‐allelic single nucleotide polymorphisms (SNPs), as described in Atwell *et al*. (2010). After quality control and imputation, this set of SNPs was reduced to a set of 214 051 SNPs. For GWA analysis, we used only SNPs with a minor allele frequency (MAF) higher than 0.05, in order to prevent spurious associations, resulting in a total of 199 360 SNPs.

### Plants, insects and pathogen

#### Plant growth conditions

*Arabidopsis* plants were grown under controlled conditions at 24 ± 1°C, 70 ± 10% relative humidity, 200 μmol m^−2^ s^−1^ photosynthetically active radiation and a diurnal cycle of 8 h : 16 h, light : dark. Seeds were vernalized at 4°C for 5 d in order to induce even germination. Plants were individually grown in 0.08‐l pots in a pasteurized (4 h, 80°C) commercial potting soil (Lentse potgrond, Lent, the Netherlands), which was mixed 1 : 1 (v/v) with autoclaved sand in Expt 1 and with pasteurized (4 h, 80°C) potting soil in Expt 2. Pots were accommodated in trays that were randomly distributed within a growth chamber. Plants were watered three times per week by adding water to the tray. Once per week, entomopathogenic nematodes were included (Entonem; http://www.koppert.nl/) to prevent infestation by fungus gnats.

#### Insect rearing

*Pieris rapae* L. (Small Cabbage White butterfly; Lepidoptera; Pieridae) was reared on Brussels sprouts plants (*Brassica oleracea* var. *gemmifera* cv Cyrus) in a growth chamber at 21 ± 1°C, 50–70% relative humidity and a diurnal cycle of 16 h : 8 h, light : dark.

*Plutella xylostella* L. (Diamondback moth; Lepidoptera; Plutellidae) was reared on Brussels sprouts plants (*B. oleracea* var. *gemmifera* cv Cyrus) in a growth chamber at 22 ± 1°C, 40–50% relative humidity and a diurnal cycle of 16 h : 8 h, light : dark.

#### Pathogen culture

The necrotrophic fungus *B. cinerea*, strain B0510 (Van der Ent *et al*., 2008), was grown on half‐strength potato dextrose agar (PDA) plates containing penicillin (100 μg ml^−1^) and streptomycin (200 μg ml^−1^) for 2 wk at room temperature. Spores were collected and re‐suspended in half‐strength potato dextrose broth (PDB; Difco Laboratories, Sparks, MD, USA) to a final density of 1.0 × 10^5^ spores ml^−1^. After a 3‐h incubation period, the spores were used for inoculation (Thomma *et al*., 1998; Pre *et al*., 2008; Van der Ent *et al*., 2008).

### Experimental design and treatments

The experimental design and treatments have been described in detail in Davila Olivas *et al*. (2016b). Briefly, two experiments were conducted. In Expt 1, we evaluated the growth of *Arabidopsis* plants after exposure to drought, herbivory by *P. rapae*, herbivory by *P. rapae* preceded by drought and herbivory by *P. rapae* preceded by *B. cinerea* infestation. The experiment was performed in 10 temporal blocks. Each block consisted of 37 randomly selected accessions plus three accessions that were present in all rounds (CS28780 (Tsu‐0), CS76113 (Col‐0) and CS76129 (Fei‐0)); the last block contained only 17 accessions. Within temporal blocks, plants were allocated in trays and the position of the tray in the rearing chamber was recorded as its position in one of the six racks, each with four shelves. The spatial location of each plant within a tray was recorded in terms of column C and row R. In each temporal block, accessions were exposed to the following five treatments: (1) no stress; (2) drought stress; (3) *P. rapae* herbivory; (4) drought and *P. rapae*; or (5) *B. cinerea* and *P. rapae*. Six replicates were included per accession and treatment combination; 11 400 plants were phenotyped in Expt 1: six replicates × 40 accessions (37 random accessions plus three accessions that were used in every temporal block) × nine temporal blocks × five treatments plus six replicates × (17 + 3 accessions) × five treatments for the last block. Plants were grown under similar conditions during the first 3 wk. Drought stress was imposed by withholding water for 7 d during the third week, whilst the rest of the plants were watered every 2 d with 1 l of water per tray. Withholding water for 7 d clearly resulted in water stress: the plants showed retarded growth and were smaller than well‐watered plants. *Botrytis cinerea* inoculation was carried out 24 h before *P. rapae* inoculation. Plants were inoculated with *B. cinerea* by pipetting 5 μl of spores suspended in half‐strength PDB (Difco Laboratories) at a concentration of 1 × 10^5^ spores ml^−1^ on two leaves of the rosette. Plants were kept at 100% relative humidity for 24 h in order to ensure successful infection by *B. cinerea*. Four‐week‐old plants were exposed to herbivory by *P. rapae* as a single or combined stress. Plants were inoculated with two newly hatched first‐instar (L1) caterpillars that were allowed to feed for 5 d. At the time of inoculation, individual plants were placed on the inverted lid of a Petri dish and the trays were filled with water to prevent caterpillars from moving between plants. Whilst the plants were on the Petri dishes, they received the same watering regime as described above; however, watering was carried out by adding 20 ml of water to each Petri dish. Rosette fresh weight (FW) was quantified for all treatments (Supporting Information Fig. S1A).

In Expt 2, we evaluated the growth reduction in *Arabidopsis* after exposure to herbivory by *P. xylostella*. The experiment was performed in four temporal blocks. Within blocks, accessions were randomly distributed over 39 trays with nine accessions per tray plus one tray that contained eight accessions. In this experiment, accession Col‐0 was included to control for a positional effect within the chamber. Each tray contained both control and treatment for Col‐0 and for nine other accessions. Plants were randomized within the trays. In each block, all accessions were phenotyped; one replicate per accession was phenotyped at a time. We repeated this four times (temporal blocks), leading to four replicates per accession. Within blocks, accessions were exposed simultaneously to either (1) no stress or (2) herbivory by *P. xylostella*; 2800 plants were phenotyped in Expt 2: four replicates (temporal blocks) × 350 accessions × two treatments. Some of the accessions displayed germination problems and so we did not have sufficient replicates for the experiment; this reduced the dataset from 350 to 321 accessions. Plants were 4 wk old when they were inoculated with two L2 larvae. Larvae were allowed to feed for 5 d. At the time of inoculation, individual plants were placed on the inverted lid of a Petri dish and the trays were filled with water to prevent caterpillars from moving between plants. Whilst the plants were on the Petri dishes, they followed the same watering regime as described above; however, watering was carried out by adding 20 ml of water to each Petri dish. Rosette FW was quantified for all treatments (Fig. S1B).

### Statistical analysis

#### Genotypic mean estimations

We obtained BLUEs (best linear unbiased estimators) for all genotype–treatment combinations as described in the literature (Jimenez‐Gomez *et al*., 2010; Filiault & Maloof, 2012; Riedelsheimer *et al*., 2012). BLUEs were estimated by a linear mixed model using the ASReml package in R (Butler *et al*., 2009).

$$\begin{array}{cc} \text{Expt}1:Y & =\mu +\text{GEN}+\text{TRT}+\text{GEN}\times \text{TRT}+\underline{\text{BLOCK}}\\ & \underline{+\text{RACK}+\text{SHELF}+\text{BLOCK}\times \text{RACK}\times }\\ & \underline{\text{SHELF}+\text{BLOCK}\times \text{RACK}\times \text{SHELF}\times \text{TRAY}+}\\ & \underline{\text{BLOCK}\times \text{RACK}\times \text{SHELF}\times \text{TRAY}\times C+}\\ & \underline{\text{BLOCK}\times \text{RACK}\times \text{SHELF}\times \text{TRAY}\times R+e}, \end{array}$$

$$\begin{array}{cc} \text{Expt}2:Y & =\mu +\text{GEN}+\text{TRT}+\text{GEN}\times \text{TRT}+\underline{\text{BLOCK}}\\ & \underline{+\text{BLOCK}\times \text{TRAY}+\text{BLOCK}\times \text{TRAY}\times C+}\\ & \underline{\text{BLOCK}\times \text{TRAY}\times R+e}, \end{array}$$

where *Y* represents the rosette FW, GEN is the genotype (accession), TRT is the treatment factor, BLOCK represents the temporal block, RACK, SHELF, TRAY, *C* and *R* are factors that represent the spatial location of the plants within the chamber, and *e* is the residual error. GEN + TRT + GEN × TRT were fitted as a fixed effect, whereas all other variables were fitted as random effects (underlined).

Using BLUEs, for each stress, we estimated the percentage difference of rosette FW relative to control plants without stress. In the treatment in which plants were exposed to both drought and herbivory by *P. rapae*, the percentage difference in rosette FW was calculated relative to plants exposed to drought. Hereafter, we refer to the percentage of biomass reduction caused by drought, *P. rapae* herbivory, *P. xylostella* herbivory, drought plus *P. rapae* and *B. cinerea* plus *P. rapae* as ‘Drought’, ‘*P. rapae*’, ‘*P. xylostella*’, ‘Drought&*Pieris*’ and ‘*Botrytis*&*Pieris*’, respectively (Table S1).

#### Data inspection

**Figure 1:**
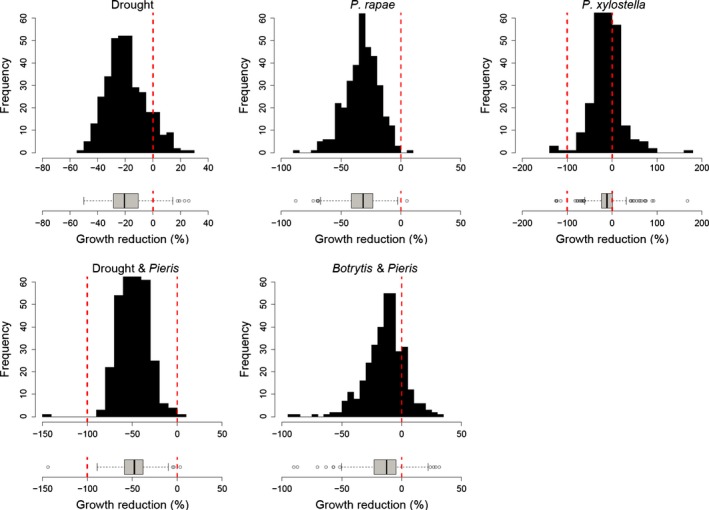
Variation in growth reduction in *Arabidopsis* plants exposed to drought, herbivory by *Plutella xylostella*, herbivory by *Pieris rapae* alone or preceded by drought or infection by the necrotrophic fungus *Botrytis cinerea*. Growth reduction was estimated from comparison with plants that had been grown without stress. For the Drought&*Pieris* treatment, growth reduction was estimated in comparison with plants exposed to drought only. Data subsets used for genome‐wide association analysis are indicated to the left of the zero and are delimited by either one or two red dashed lines. Box plots represent median value (thick line), the first plus third quartiles (box) and the lowest and highest values (whiskers); the circles represent outliers.

We initially inspected the variation in response to each stress (Fig. 1). We observed that some accessions had larger biomass under treatment than under control conditions. We reasoned that these accessions displayed tolerance to the treatment. Because tolerance and resistance traits have a different genetic basis (Strauss & Agrawal, 1999; Carmona *et al*., 2011; Karinho‐Betancourt & Nunez‐Farfan, 2015), we only included data for accessions displaying a reduction in biomass under the treatment compared with control conditions (Table S1). This dataset was used for all downstream analyses.

#### Phenotypic and genotypic correlations

Phenotypic correlations were estimated by Spearman correlation of the genotypic mean BLUEs for every possible combination of two traits. Spearman correlation analyses were implemented in the package Hmisc in R (Harrell, 2009). Genetic correlations reflect the overlap in polygenic effects amongst traits. A perfect genetic correlation (*r*_g_ = 1) between two traits indicates that exactly the same loci control these traits, and a non‐perfect genetic correlation (*r*_g_ < 1) reveals a mixture of unique and common genetic effects among traits. Genetic correlations were estimated according to the multi‐trait mixed model described in Korte *et al*. (2012).

#### Narrow‐sense heritability

Phenotypic variance can be decomposed into variance caused by genetic and environmental factors. Broad‐sense heritability (*H* ^2^) estimates the proportion of phenotypic variance that is caused by genetic factors. Genetic variance can be a result of additive, dominant or epistatic effects. Narrow‐sense heritability (*h* ^2^) captures the proportion of genetic variance that is caused by additive genetic effects. Narrow‐sense heritability is important because it is an indicator of how a population responds to artificial or natural selection (Wray & Visscher, 2008). Narrow‐sense heritability estimates for each response were estimated with the heritability package in R (Kruijer *et al*., 2015).

### Genome‐wide association analysis

Variation in growth reduction under different stresses was linked to regions in the genome that explained the observed variation using a GWA analysis, carried out employing Fast‐LMM software, as described in Cao *et al*. (2011). Fast‐LMM assumes the following mixed model for each SNP:

y=μ+Xβ+g+e

where *y* is a vector of *n* phenotype values. *X* is a design matrix in which trait means are included with other fixed effects. In *X*, β is the effect of the Col‐0 allele. $g∼N(0\sigma_{g}^{2}K)$ and $e∼N(0\sigma_{e}^{2}I)$ are random effects. We tested the hypothesis β *=* 0 using generalized least squares (GLS), conditional on residual maximum likelihood (REML) estimates $\sigma_{g}^{2}$ and $\sigma_{e}^{2}$ for the genetic and environmental variance. The proportion of the genetic variance explained by each SNP was estimated using two methods: (1) the $R_{\mathrm{LR}}^{2}$ statistic proposed by Cox & Snell (1989), which is 1 − exp(−(2/*n*)(*L*_1_ − *L*_0_)); and (2) 2 × (β^2^ *p*(1 − *p*)/σ^2^), where β is the allele effect*, p* is the frequency and σ^2^ is the sample variance. Fast‐LMM corrects for population structure using a GRM (genetic relatedness matrix) instead of a kinship matrix as in Emmax software (Kang *et al*., 2010). Fast‐LMM is considered to be more powerful than Emmax because: (1) each SNP test is based on a local kinship matrix that consists of the GRM based on all markers, except those that are in a window of 20 kb on each side of the tested SNP; and (2) the genetic and residual variance components are estimated for each SNP, instead of assuming that these are constant across the genome. In order to reduce the amount of spurious associations caused by rare variants, a MAF of 5% was used.

#### Candidate gene selection

For the selection of candidate genes, an arbitrary threshold of −log_10_(*P*) ≥ 4 was considered. The same threshold has been used in other GWA studies on *A. thaliana* (Verslues *et al*., 2014; El‐Soda *et al*., 2015; Van Rooijen *et al*., 2015; Kooke *et al*., 2016). Regions containing SNPs with −log_10_(*P*) ≥ 4 were considered for further analysis as described by El‐Soda *et al*. (2015). A search window was defined by SNPs in LD (LD ≥ 0.5, if no SNPs were found at 0.5 the threshold was lowered to 0.4) in a window ± 20 kb with significant SNPs. SNPs in LD from the 250K array were enriched with SNPs in LD from 1001 genomes (http://1001genomes.org/), as described in Bac‐Molenaar *et al*. (2015). Thus, a search window was defined by the first and last SNP in LD. All genes within a search window were considered to be potential candidate genes. To narrow down the list of candidate genes, further analyses were performed. First, gene annotation from candidate genes was obtained from TAIR 10. Furthermore, candidate genes were enriched with gene expression data from different sources. Data from tissue exposed to the phytohormones JA, ABA or ET were obtained from a public database (http://bar.utoronto.ca/) (Toufighi *et al*., 2005). Expression data for *A. thaliana* plants infested with *P. xylostella* were obtained from Ehlting *et al*. (2008). RNA‐sequencing (RNA‐seq)‐based expression data for *A. thaliana* plants infested with *P. rapae*, drought and *P. rapae*, and *B. cinerea* and *P. rapae* were obtained from Davila Olivas *et al*. (2016a). RNA‐seq‐based expression data for drought responses were obtained from P. Huang *et al*. (unpublished). The data are summarized in Tables S2–S6.

## Results

### Variation within and between responses of *A*. *thaliana* to single or multiple stresses

We observed extensive variation among the accessions in the percentage of growth reduction for plants exposed to the different stresses addressed in this study (Fig. 1; Table 1). The largest variation was observed for the response to *P. xylostella* (CV = 78%), whereas the lowest variation was observed for the response to Drought&*Pieris* (CV = 31%) (Table 1). Narrow‐sense heritability estimates ranged from 0.17 to 0.52 (Table 1). No relationship between narrow‐sense heritability and variation in stress responses was observed (Table 1).

**Table 1:** Summary of variation in the percentage of biomass reduction of 350 *Arabidopsis thaliana* accessions on exposure to drought, herbivory by *Plutella xylostella* and herbivory by *Pieris rapae* alone or preceded by drought or infection with the necrotrophic fungus *Botrytis cinerea*

Trait	Min.	Mean	Max.	*n*	CV	*h* ^2^	CI	va	ve
*Botrytis*&*Pieris*	0.08	18.88	90.13	285	74	0.52	0.17–0.86	103.80	94.77
*P. rapae*	2.67	32.62	87.80	345	42	0.51	0.22–0.79	96.96	93.20
*P. xylostella*	0.03	21.66	82.22	234	78	0.42	0.13–0.78	121.71	166.41
Drought	0.29	22.67	50.07	307	48	0.42	0.15–0.76	49.99	68.16
Drought&*Pieris*	3.62	48.29	89.06	344	31	0.17	0.03–0.59	36.08	181.48

Traits are ordered by narrow‐sense heritability. Min., lowest value; Max., highest value; *n*, number of accessions analysed; CV, coefficient of variation (%); *h* ^2^, narrow‐sense heritability; CI, heritability 95% confidence intervals; va, additive genetic variance; ve, residual variance.

### Genetic and phenotypic correlations among traits

To explore the relationships among different traits, we performed Spearman correlation analysis on the phenotypic values (Table 2). The response to drought displayed a negative correlation with the other traits. Furthermore, the largest phenotypic correlation was observed between the responses to *Botrytis*&*Pieris* and *P. rapae* (ρ = 0.52). A low phenotypic correlation was observed between the responses to *P. xylostella* and *P. rapae* (ρ = 0.15). Because phenotypic correlations may arise as a result of genetic and environmental factors, a better estimate of shared genetic basis between traits is genetic correlation. The largest genetic correlation was between the responses to *Botrytis*&*Pieris* and to *P. rapae* (*r*_g_ = 0.98), followed by the responses to drought and to *Botrytis*&*Pieris* (*r*_g_ = −0.81).

**Table 2:** Phenotypic and genetic correlations among the percentages of biomass reduction in plants exposed to drought, herbivory by *Plutella xylostella* and herbivory by *Pieris rapae* alone or preceded by drought or infection with the necrotrophic fungus *Botrytis cinerea*

Trait	Drought	*P. rapae*	Drought&*Pieris*	*Botrytis*&*Pieris*	*P. xylostella*
Drought		−0.65	NC	−0.89	−0.42
*P. rapae*	−0.25		NC	0.98	0.20
Drought&*Pieris*	−0.38	0.48		NC	0.64
*Botrytis*&*Pieris*	−0.29	0.53	0.40		0.33
*P. xylostella*	**−0.12**	**0.15**	**0.16**	**0.14**	

NC, residual maximum likelihood did not converge. Phenotypic correlations (Spearman correlation coefficients) are indicated below the diagonal. Genetic correlations were estimated by residual maximum likelihood as in Korte *et al*. (2012). Genetic correlation estimates (*r*_g_) are indicated above the diagonal. Values below the diagonal that were not significant (*P *>* *0.05 after Bonferroni correction) are indicated in bold.

### Genetic architecture underlying variation in responses to single and multiple stresses

**Figure 2:**
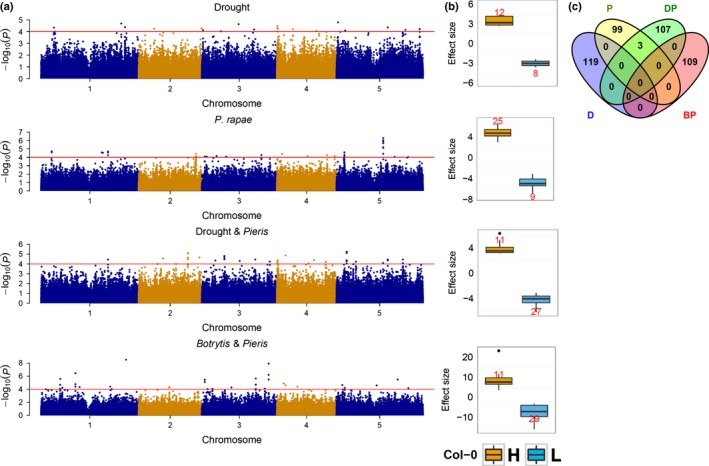
Genome‐wide association analysis of growth reduction in plants exposed to a single stress imposed by drought or herbivory by *Pieris rapae* or multiple stresses imposed by *P. rapae* preceded by drought or infection by the necrotrophic fungus *Botrytis cinerea*. (a) Manhattan plots. Red line indicates an arbitrary threshold set at −log_10_(*P*) ≥ 4, as described in the Materials and Methods section for the detection of significant associations. (b) Effect sizes for significant single nucleotide polymorphisms (SNPs). Effect sizes are indicated for the Col‐0 allele. Yellow and blue indicate a higher and lower reduction in biomass associated with the Col‐0 allele, respectively. The number of significant SNPs is indicated in red. Box plots represent the median value (thick line), the first plus third quartiles (box) and the lowest and highest values (whiskers); the circles represent outliers. (c) Candidate genes. Genes in a 20‐kb window of a significant SNP were considered as candidates. D, Drought; P, *Pieris rapae*; DP, Drought&*Pieris*; BP,* Botrytis*&*Pieris*.

To obtain insight into the genetic architecture underlying the variation in responses to single stresses imposed by drought or *P. rapae* feeding and the two multiple stress situations, Drought&*Pieris* and *Botrytis*&*Pieris*, we performed a GWA analysis. We used a threshold of −log_10_(*P*) ≥ 4 to declare an SNP being associated with a trait. SNPs in LD were considered in a region of ± 20 kb from a significant SNP (Tables S2–S5). A summary of the GWA analysis for each trait is presented in Fig. 2 and Table 3. For the responses to single stresses, the numbers of significant SNPs amounted to a total of 20 (64 SNPs in LD) for the response to drought and 34 (78 SNPs in LD) for the response to *P. rapae*. For the responses to combined stresses, the numbers of significant SNPs were greater than in the responses to single stress situations: 38 (106 SNPs in LD) for the response to Drought&*Pieris* and 40 (106 SNPs in LD) for the response to *Botrytis*&*Pieris*. Effect sizes for the Col‐0 allele were estimated for each trait. For most of the traits, the significant SNPs displayed low effect sizes, except for the response to *Botrytis*&*Pieris* (Fig. 2b; Tables S2–S5). The response to drought displayed the lowest effect sizes, ranging from −4 (meaning that accessions with the Col‐0 allele have 4% less biomass reduction than accessions carrying the alternative allele) to 4. The response to *Botrytis*&*Pieris* displayed the highest effect sizes, ranging from −16 to 23. For most of the traits, the significant SNPs explained a low percentage of the genetic variance (Tables S2–S5). The maximum percentage of genetic variance explained by an SNP for the response to each stress was 7% for drought, 7% for *P. rapae*, 5% for Drought&*Pieris* and 12% for *Botrytis*&*Pieris*. Despite the moderate to high genetic correlations among traits, little overlap was observed between the significant SNPs, between the regions delimited by SNPs in LD (QTLs), and therefore also between the genes contained within QTLs (Fig. 2c).

**Table 3:** Summary of genome‐wide association analysis per trait

Trait	SNPs	SNPs in LD^a^	Strings	Singletons	QTLs	Genes^b^
Drought	20	64	12	6	18	119
*P. rapae*	34	78	13	5	18	102
Drought&*Pieris*	38	106	13	6	19	110
*Botrytis*&*Pieris*	40	106	16	9	25	109
*P. xylostella*	57	238	22	10	32	141

a Single nucleotide polymorphisms (SNPs) in linkage disequilibrium (LD) ≥ 0.5 were considered in a region of ± 20 kb from a significant SNP. Numbers of SNPs in LD are based on the 250K SNP array.

b A search window was defined taking into consideration additional SNPs in LD from the 1001 genomes project (see the Materials and Methods section). All genes within a search window were considered as candidate genes.

QTL, quantitative trait locus.

### Differences in genetic architecture underlying responses to two specialist insect herbivores

**Figure 3:**
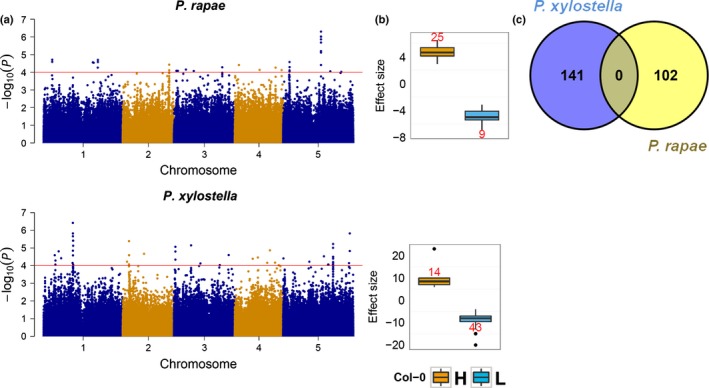
Genome‐wide association analysis of growth reduction in plants exposed to herbivory by the specialist insects *Pieris rapae* and *Plutella xylostella*. (a) Manhattan plots. Red line indicates an arbitrary threshold set at −log_10_(*P*) ≥ 4, as described in the Materials and Methods section for the detection of significant associations. (b) Effect sizes for significant single nucleotide polymorphisms (SNPs). Effect sizes are indicated for the Col‐0 allele. Yellow and blue indicate a higher and lower reduction in biomass associated with the Col‐0 allele, respectively. Number of significant SNPs is indicated in red. Box plots represent the median value (thick line), the first plus third quartiles (box) and the lowest and highest values (whiskers); the circles represent outliers. (c) Candidate genes. Genes in a 20‐kb window of a significant SNP were considered as candidates.

We also investigated the genetic architecture of *A. thaliana's* response to *P. xylostella* and compared it with the genetic architecture of the response to *P. rapae*. We identified a larger number of significant SNPs for the response to *P. xylostella* (57 SNPs plus 238 SNPs in LD) than for the response to *P. rapae* (34 SNPs plus 78 SNPs in LD) (Fig. 3; Table 3). Furthermore, the effect size of SNPs associated with the response to *P. xylostella* (from −20 to 22) was larger than that for the response to *P. rapae* (from −7 to 7) (Fig. 3b). The maximum percentage of genetic variance explained by the SNPs associated with the response to *P. xylostella* was 10%, whereas, for the response to *P. rapae*, it was 7% (Table S3, S6). No common significant SNPs, regions delimited by SNPs in LD (QTLs) and therefore also genes contained within QTLs were observed between *P. xylostella* and *P. rapae* (Fig. 3c).

### Candidate genes for drought resistance

Eighteen QTLs were identified for biomass reduction in response to drought. Within these regions, several genes that are known to play a role in drought acclimation were identified. For example, QTL 7 on chromosome 3 contained only one gene, *AT3G17520,* which encodes a late embryogenesis‐abundant protein (LEA protein). In general, LEA proteins have been suggested to play a protective role for other proteins under conditions of water stress in vegetative tissues (Battaglia *et al*., 2008). The closest significant SNP (Chr3: 5997119) explained 5% of the phenotypic variance; the Col‐0 allele was rare (86 accessions including Col‐0) and was associated with a greater reduction in *A. thaliana* FW (Table S2; Fig. S2). This gene was induced on drought stress and ABA application (Table S2). This suggests that this may be the causal gene for QTL 7.

### Candidate genes involved in plant–insect interactions

We analysed the variation in growth reduction of *Arabidopsis* in response to two specialist insect herbivores, *P*. *rapae* and *P*. *xylostella*. GWA allowed the linking of this variation to several regions in the plant genome. We identified a total of 18 and 32 QTLs for the responses to *P*. *rapae* and *P*. *xylostella*, respectively (Table 3). Within these regions, several candidate genes with a known function in plant resistance against insect herbivores were identified.

For *P*. *rapae*, QTL 15 on chromosome 5 contained *AT5G07690* (*MYB29*) and AT5G0700 (*MYB76*) (Table S3). The closest significant SNP (Chr5: 2454480) explained 4% of the phenotypic variance. The Col‐0 allele was rare (55 accessions including Col‐0) and was associated with a greater reduction in *A*. *thaliana* FW (Table S3; Fig. S3). Both genes were induced in response to *P*. *rapae* infestation. Furthermore, *MYB76* was induced by JA and ET treatment (Table S3).

Another interesting QTL for the response to *P*. *rapae* was QTL 1 on chromosome 1, which contained *AT1G10060* (*BCAT‐1*) and *AT1G10070* (*BCAT‐2*) (Table S3). The closest significant SNP (Chr1: 3294935) explained 5% of the phenotypic variance; the Col‐0 allele was rare (89 accessions including Col‐0) and was associated with a greater reduction in *A*. *thaliana* FW (Table S3; Fig. S3). Furthermore, both genes were induced by *P*. *rapae* infestation and application of the phytohormones JA and ABA (Table S3).

For the response to *P*. *xylostella,* more QTLs were identified than for the response to *P*. *rapae* (Table 3). QTL 18 on chromosome 4 contained only two genes, *AT4G11310* (*CP1*) and *AT4G11320* (*CP2*). The closest significant SNP (Chr4: 3294935) explained 7% of the phenotypic variance; the Col‐0 allele was common (159 accessions including Col‐0) and was associated with a smaller reduction in *A*. *thaliana* FW (Table S6; Fig. S4). *CP1* and *CP2* were induced by both *P*. *rapae* and *P*. *xylostella* infestation. In addition, they were also induced by JA application (Table S6). Both *CP1* and *CP2* encode CYSTEINE PROTEASE enzymes (TAIR 10). CP2 has been implicated in increasing the resistance of cotton against *Helicoverpa armigera* (Mao *et al*., 2013).

Another example is QTL 32 on chromosome 5 which contains *AT5G64080* (*XYP1*). The closest significant SNP (Chr5: 25640504) explained 9% of the phenotypic variance; the Col‐0 allele was common (178 accessions including Col‐0) and was associated with a smaller reduction in *A*. *thaliana* FW (Table S6; Fig. S4). *XYP1* was induced by *P*. *rapae* and *P*. *xylostella* infestation (Table S6), and encodes a proteinase inhibitor/seed storage/lipid transfer protein. This type of protein has been implicated in anti‐nutritional defences against insect herbivores (Heidel‐Fischer *et al*., 2014).

### Candidate genes for combined stresses

Nineteen and 25 QTLs were identified for the responses to the combined stresses Drought&*Pieris* and *Botrytis*&*Pieris*, respectively (Table 3). QTL 1 for Drought&*Pieris* and QTL 3 for *P. rapae* on chromosome 1 overlapped to some extent. The significant SNPs associated with each QTL were different, but the QTLs overlapped by SNPs in LD. The Col‐0 allele for significant SNPs was rare and was associated with a greater reduction in *A. thaliana* FW (Tables S3, S4; Figs S3, S5). *AT1G55740* (*SIP1*) and *AT1G55760* within this QTL displayed interesting expression patterns. *SIP1* was induced by *P. rapae* infestation, drought and ABA application. *AT1G55760* was induced by drought and ABA, but was repressed by JA application (Table S3, S4).

For the response to Drought&*Pieris*, QTL 10 on chromosome 4 and QTL 19 on chromosome 5 contained the bHLH transcription factors *AT4G00480* (*MYC1*) and *AT5G50915*. The Col‐0 allele in both QTLs was rare and was associated with a smaller reduction in *A. thaliana* FW. Both genes were induced by *P. rapae* infestation and slightly induced by drought (Table S4). Natural variation in trichome density in *A. thaliana* has been associated with genetic variation in *MYC1* (Symonds *et al*., 2011). Several other bHLH transcription factors (e.g. *MYC2*,* MYC3*,* MYC4*,* MYC5*) are well established in the literature as major regulators of JA‐ and ABA‐mediated responses, insect resistance and drought responses (Dombrecht *et al*., 2007; Shinozaki & Yamaguchi‐Shinozaki, 2007; Schweizer *et al*., 2013; Li *et al*., 2014; Qi *et al*., 2015). QTLs containing bHLH transcription factors were also identified for the responses to *P. rapae* (*AT1G51140*) and *P. xylostella* (*AT1G12540*) (Tables S3, S6).

For the response to *Botrytis*&*Pieris*, no bHLH transcription factors were identified. However, QTL 3 on chromosome 1 contained *AT1G19210*, an ERF/AP2 transcription factor (Table S5). The significant SNP with the highest effect within this QTL (Chr1: 6627245) explained 6% of the phenotypic variance; the Col‐0 allele was common (232 accessions including Col‐0) and was associated with a smaller reduction in *A. thaliana* FW (Table S5; Fig. S6). *AT1G19210* was induced on *P. rapae* infection, drought, JA, ABA and ET application (Table S5). Several homologues of *AT1G19210* (e.g. *RAP2.1*,* RAP2.9*,* RAP2.10*) have been implicated in tolerance to drought and freezing and resistance to necrotrophic fungi (Tsutsui *et al*., 2009; Dong & Liu, 2010).

## Discussion

### Genetic architecture of *A*. *thaliana* resistance to specialist insects

In this study, we have analysed the genetic architecture of *A. thaliana* responses to *P. xylostella* and *P. rapae*, two insect species specialized on the mustard family (Brassicaceae). We identified variation in resistance to both insect herbivores among *A. thaliana* accessions that is genetically determined, as indicated by the moderate narrow‐sense heritability estimates for the responses to both species (Table 1). Heritability estimates reported for resistance to generalist insects are higher than for specialist insects (Jander *et al*., 2001; Kliebenstein *et al*., 2002). For example, in the latter study, using two RIL populations, broad‐sense heritability estimates for resistance to the generalist *Trichoplusia ni* ranged from 0.26 to 0.31, whereas, for resistance to the specialist insect *P. xylostella*, it ranged from 0.12 to 0.18 (Kliebenstein *et al*., 2002). Although resistance to generalists is controlled by QTLs of large effect, resistance to specialists seems to be under the control of QTLs of small effect (Jander *et al*., 2001; Kliebenstein *et al*., 2002; Pfalz *et al*., 2007). Several studies have reported QTLs associated with insect resistance, but few have identified the causal loci (Jander *et al*., 2001; Pfalz *et al*., 2007; Ordas *et al*., 2009; Schranz *et al*., 2009; Prasad *et al*., 2012). The QTLs identified in this study for both insect species had small effects on plant phenotype (Tables S3, S6). However, none of the QTLs identified here were shared for resistance to the two specialist insect herbivores (Fig. 3; Table 3), suggesting that the resistance mechanisms are species specific. Similar results were obtained in a QTL study using *P. brassicae* and *P. xylostella* and *A. thaliana*, where no common QTLs were identified (Pfalz *et al*., 2007). Furthermore, microarray analyses have revealed that *P. rapae* and *P. xylostella* elicit different transcriptomic responses in *A. thaliana*, supporting the notion of species‐specific mechanisms of resistance (Ehlting *et al*., 2008; Bidart‐Bouzat & Kliebenstein, 2011).

QTL analyses in *A*. *thaliana* and other species in the Brassicaceae have identified several genes involved in the metabolism of glucosinolates as the source of resistance to generalist insects (Jander *et al*., 2001; Kliebenstein *et al*., 2002; Schranz *et al*., 2009). However, specialist insects, such as *P*. *rapae* and *P*. *xylostella*, have developed distinct detoxification mechanisms rendering glucosinolates ineffective (Schoonhoven *et al*., 2005; Wheat *et al*., 2007; De Vos *et al*., 2008; Müller *et al*., 2010; Agrawal *et al*., 2012). Interestingly, one of the QTLs identified here for resistance to *P*. *rapae* contained, as the most likely candidates, *MYB29* and *MYB76,* encoding for two transcription factors involved in the induced production of aliphatic glucosinolates (Hirai *et al*., 2007). Indeed, the double mutant *myb29myb28*, which lacks aliphatic glucosinolates, is less preferred for feeding by *P*. *rapae* than is Col‐0 (Müller *et al*., 2010). Another QTL, identified for the response to *P*. *rapae*, contained, as most likely candidates, *BCAT‐1* and *BCAT‐2*, which are enzymes involved in branched amino acid (leucine (Leu), valine (Val) and isoleucine (Ile)) (BCAA) metabolism (Diebold *et al*., 2002). Interestingly, homologues of these genes (*BCAT‐3*,* BCAT‐4*,* BCAT‐6*) have been implicated in the production of aliphatic glucosinolates (Schuster *et al*., 2006; Lachler *et al*., 2015). Furthermore, co‐expression networks have revealed that *BCAT‐4* is co‐expressed with *MYB29*,* MYB28* and several putative genes involved in Leu metabolism (Hirai *et al*., 2007). Interestingly, an evolutionary link has been suggested between aliphatic glucosinolates and BCAA metabolism (Schuster *et al*., 2006). In *Boechera stricta*, a species related to *A*. *thaliana*, QTL analysis identified a QTL that controls variation in allocation between methionine and BCAA‐derived glucosinolates and resistance to the generalist caterpillar *T*. *ni* (Schranz *et al*., 2009).

For *P. xylostella,* a small‐effect QTL near *ERECTA* on chromosome 2 has been reported in *Arabidopsis* and *B. oleracea* (Kliebenstein *et al*., 2002; Ramchiary *et al*., 2015). We identified two QTLs on chromosome 2. However, neither of these was in the vicinity of *ERECTA*.

### Genetic architecture of resistance against multiple stresses

In complex environments, such as natural and agricultural ecosystems, plants experience several stresses that co‐occur (Mittler & Blumwald, 2010; Chan *et al*., 2011; Prasch & Sonnewald, 2013; Rasmussen *et al*., 2013; Kissoudis *et al*., 2014). Here, we compared the genetic architecture of the combined stresses imposed by drought plus *P. rapae* or *B. cinerea* plus *P. rapae* with the single stress imposed by *P. rapae* alone. We observed genetically determined variation for both combined stresses, as indicated by their narrow‐sense heritability values (Table 1). However, although the total phenotypic variance for resistance to drought plus *P. rapae* was larger than for the single stress situation, the proportion that was explained by genetic factors was dramatically lower (Table 1). This implies that there is little genetic variation for this trait, and this may have implications for the power of GWA analysis to identify true associations with this trait. Only a few studies have conducted QTL analysis on plant responses to combined stresses, and some have identified similar caveats. For example, in a study conducted in a maize population, a lower genetic variance was observed under a combination of drought plus heat than in the single stress situations (Cairns *et al*., 2013). Furthermore, in a tomato population exposed to a combination of salt and powdery mildew, a reduction in phenotypic variation in disease resistance was observed under combined stress in comparison with the single stress situation (Kissoudis *et al*., 2015). The low heritability and phenotypic variation under combined stresses may represent a pitfall for QTL identification and breeding for combined stresses.

However, for the combined stresses of *B. cinerea* plus *P. rapae*, no difference in narrow‐sense heritability was observed compared with the single stress imposed by *P. rapae* (Table 1). Furthermore, both traits displayed a high level of genetic correlation, suggesting that common genes influence both traits (Table 2). Despite the high genetic correlation between the response to *B. cinerea* plus *P. rapae* vs the response to the single stress *P. rapae*, no common QTLs were identified (Table 3; Fig. 2). It may be that the QTLs that underlie the similarity of both traits are QTLs of small effect that were not identified at the threshold used in this study. An alternative tool that may help to unravel the genetic commonality between these two traits is a multi‐trait GWA that allows for the identification of SNPs with common and opposite effects among highly correlated traits (Korte *et al*., 2012). This may increase the power of univariate GWAs for highly correlated traits (Korte *et al*., 2012).

Contrary to the limited overlap found between QTLs identified for combined stresses (Table 3; Fig. 2), other studies have identified a mixture of novel QTLs and QTLs that are present in the single stress case (Cairns *et al*., 2013; Makumburage *et al*., 2013). However, the effect of QTLs under stress combinations was never observed to be in the same direction as in the single stress case (Makumburage *et al*., 2013). Thus, the genetic architecture underlying single and combined stresses appears to be different. In addition to the few studies addressing QTL identification, several papers have addressed whole transcriptome changes in response to combinations of stresses (Atkinson *et al*., 2013; Prasch & Sonnewald, 2013; Rasmussen *et al*., 2013). These studies concluded that the transcriptional response to combined stresses was different from the single stress situation. Furthermore, up to 60% of the transcriptional changes in response to combined stresses could not be predicted from the response to each individual stress (Rasmussen *et al*., 2013).

Despite co‐occurrence being the rule rather than the exception under natural conditions, the importance of studying stress combinations has only just started to be acknowledged by the scientific community. It may be that the complexity of the experimental design, the number of possible stress combinations and the complex logistics have held back the adoption of this type of experiment. The present study, together with several studies on QTL mapping and transcriptomic changes under combinations of stresses, have concluded that responses to combined stresses cannot be predicted from the responses to individual stresses (Voelckel & Baldwin, 2004). This further underlines the complexity of the events that take place when plants are challenged by combinations of stresses, and highlights the importance of studying combinations of stresses in addition to studies of single stresses.

Finally, *P. rapae* and *P. xylostella* are major pests on *Brassica* crops, such as cabbage and broccoli (Agrawal & Kurashige, 2003; Zalucki *et al*., 2012). A good understanding of genetic architecture and the unequivocal identification of genes underlying variation in resistance will benefit the breeding process of cultivars that are more resistant to these insect pests.

In this study, we identified several candidate genes for resistance to two specialist insects (*P. rapae* and *P. xylostella*), an abiotic stress (drought) and two combined stresses (drought plus *P. rapae*;* B. cinerea* plus *P. rapae*). We have provided evidence using transcriptomic data from independent studies which show that these genes are differentially expressed when plants are exposed to the same stresses as addressed in this study. The genes identified here remain to be validated by, for example, allelic complementation or mutant experiments in future studies. However, it should be realized that a mutant effect is estimated in just one genetic background and tests all genetic (additive, dominance, epistatic) and genotype‐by‐environment effects for that position/gene simultaneously. A QTL allele effect in an association analysis represents the conditional difference between two groups of genotypes with alternative versions of an SNP, and so the additive effect in a more general sense than the additive effect in the mutant, where these groups are part of an association panel. Moreover, the data probably deal with quantitative phenotypic variation and small QTL effects; knockout mutants do not provide the best means to confirm such QTLs, as mutants are especially suitable for genes and QTLs with a qualitative effect. The present study, whilst revealing the genetic architecture underlying resistance to several environmental stresses, also highlights the complexity of studying combinations of stresses.

## Author contributions

N.H.D.O., J.J.A.v.L., G.G. and M.D. planned and designed the research. N.H.D.O., C.L.W. and W.K. performed the experiments and/or analysed the data. N.H.D.O., J.J.A.v.L. and M.D. executed data interpretation. N.H.D.O., W.K., J.J.A.v.L. and M.D. wrote the manuscript.

## Supporting information

Please note: Wiley Blackwell are not responsible for the content or functionality of any Supporting Information supplied by the authors. Any queries (other than missing material) should be directed to the *New Phytologist* Central Office.

**Fig. S1** Time scheme of treatments.
**Fig. S2** Single nucleotide polymorphisms (SNPs) associated with reduction in *Arabidopsis thaliana* rosette fresh weight in response to drought.
**Fig. S3** Single nucleotide polymorphisms (SNPs) associated with reduction in *Arabidopsis thaliana* rosette fresh weight in response to *Pieris rapae*.
**Fig. S4** Single nucleotide polymorphisms (SNPs) associated with reduction in *Arabidopsis thaliana* rosette fresh weight in response to *Plutella xylostella*.
**Fig. S5** Single nucleotide polymorphisms (SNPs) associated with reduction in *Arabidopsis thaliana* rosette fresh weight in response to drought plus *Pieris rapae*.
**Fig. S6** Single nucleotide polymorphisms (SNPs) associated with reduction in *Arabidopsis thaliana* rosette fresh weight in response to *Botrytis cinerea* plus *Pieris rapae*.

**Table S1** Percentage reduction in rosette fresh weight in *Arabidopsis thaliana* on stress exposure

**Table S2** Candidate genes for reduction in *Arabidopsis thaliana* rosette fresh weight in response to drought

**Table S3** Candidate genes for reduction in *A. thaliana* rosette fresh weight in response to *Pieris rapae*

**Table S4** Candidate genes for reduction in *Arabidopsis thaliana* rosette fresh weight in response to drought plus *Pieris rapae*

**Table S5** Candidate genes for reduction in *Arabidopsis thaliana* rosette fresh weight in response to *Botrytis cinerea* plus *Pieris rapae*

**Table S6** Candidate genes for reduction in *Arabidopsis thaliana* rosette fresh weight in response to *Plutella xylostella*
