# Supplementary material for: Genome‐wide association analysis reveals distinct genetic architectures for single and combined stress responses in Arabidopsis thaliana
Source: New Phytol. 2016 Sep 8;213(2):838–51. doi: 10.1111/nph.14165 (PMC5217058; doi:10.1111/nph.14165)
Supplement: Supplementary file 1 — Fig. S1 Time scheme of treatments. Fig. S2 Single nucleotide polymorphisms (SNPs) associated with reduction in Arabidopsis thaliana rosette fresh weight in response to drought. Fig. S3 Single nucleotide polymorphisms (SNPs) associated with reduction in Arabidopsis thaliana rosette fresh weight in response to Pieris rapae. Fig. S4 Single nucleotide polymorphisms (SNPs) associated with reduction in Arabidopsis thaliana rosette fresh weight in response to Plutella xylostella. Fig. S5 Single nucleotide polymorphisms (SNPs) associated with reduction in Arabidopsis thaliana rosette fresh weight in response to drought plus Pieris rapae. Fig. S6 Single nucleotide polymorphisms (SNPs) associated with reduction in Arabidopsis thaliana rosette fresh weight in response to Botrytis cinerea plus Pieris rapae. [file NPH-213-838-s001.pdf]

New Phytologist

***New Phytologist* Supporting Information**

Article title: Genome-wide association analysis reveals distinct genetic architectures for single and combined stress responses in *Arabidopsis thaliana*

Authors: Nelson H. Davila Olivas, Willem Kruijer, Gerrit Gort, Cris L. Wijnen, Joop J.A. van Loon and Marcel Dicke

Article acceptance date: 11 July 2016

The following Supporting Information is available for this article:

Figs S1–S6 included below; see separate Excel files for **Tables S1–S6**.

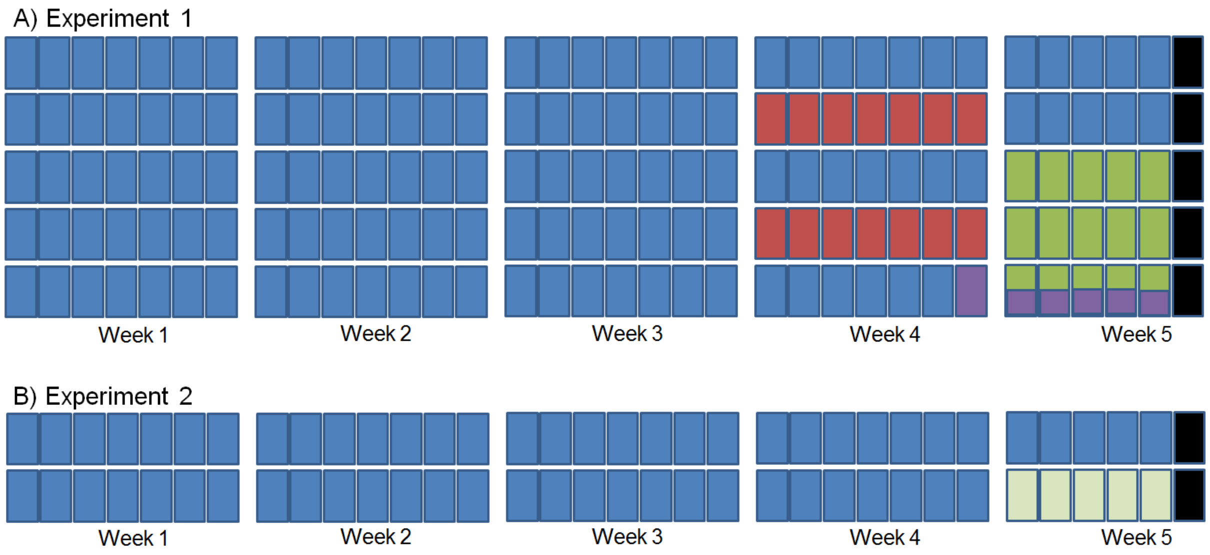

Fig. S1 Treatments time scheme. (A) In Experiment 1 the effects of drought and herbivory by *P. rapae* caterpillars either as single treatment or preceded by drought stress or *B. cinerea* infestation were evaluated. Blue indicates that the plants were growing without stress. Drought stress is indicated in red. Time of inoculation of *B. cinerea* is indicated in purple. Time of infestation with *P. rapae* is indicated in dark green. Measurement time is indicated in black. (B) In Experiment 2 the effect of herbivory by *P. xylostella* caterpillars was evaluated. Blue indicates that the plants were growing without stress. Time of infestation with *P. xylostella* is indicated in light green. Measurement time is indicated in black.

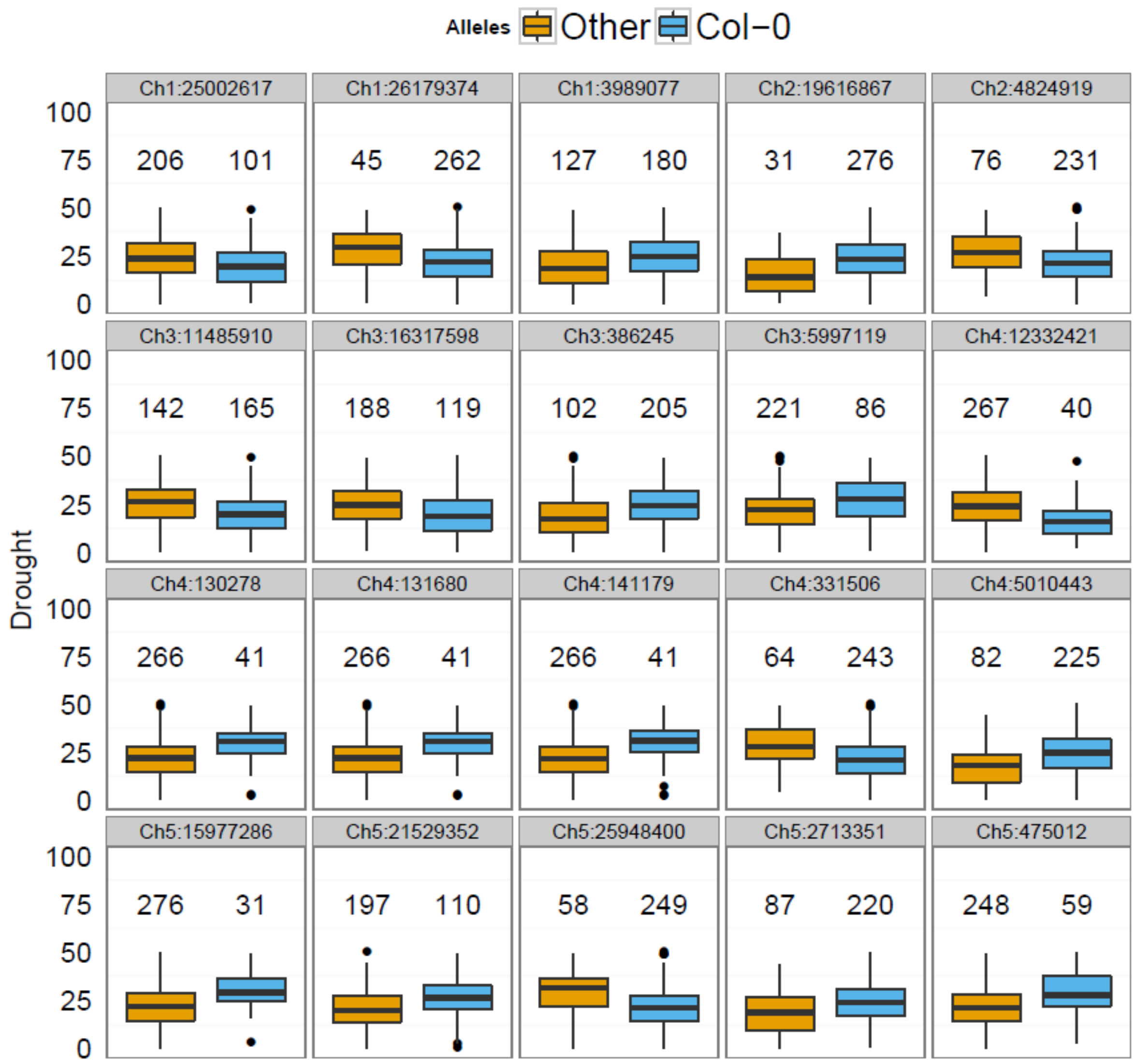

Fig. S2 Allele effects of significant SNPs associated with *Arabidopsis thaliana* rosette fresh weight reduction in response to drought. Chromosome and SNP position are indicated. Number of accessions having the Col-0 allele or another allele are indicated in above the boxes. *Y*-axis represents the percentage of biomass reduction compared to the control situation. Box plots represent median value (thick line), the first plus third quartiles (box), and the whiskers represent the lowest and highest values; the circles represent outliers.

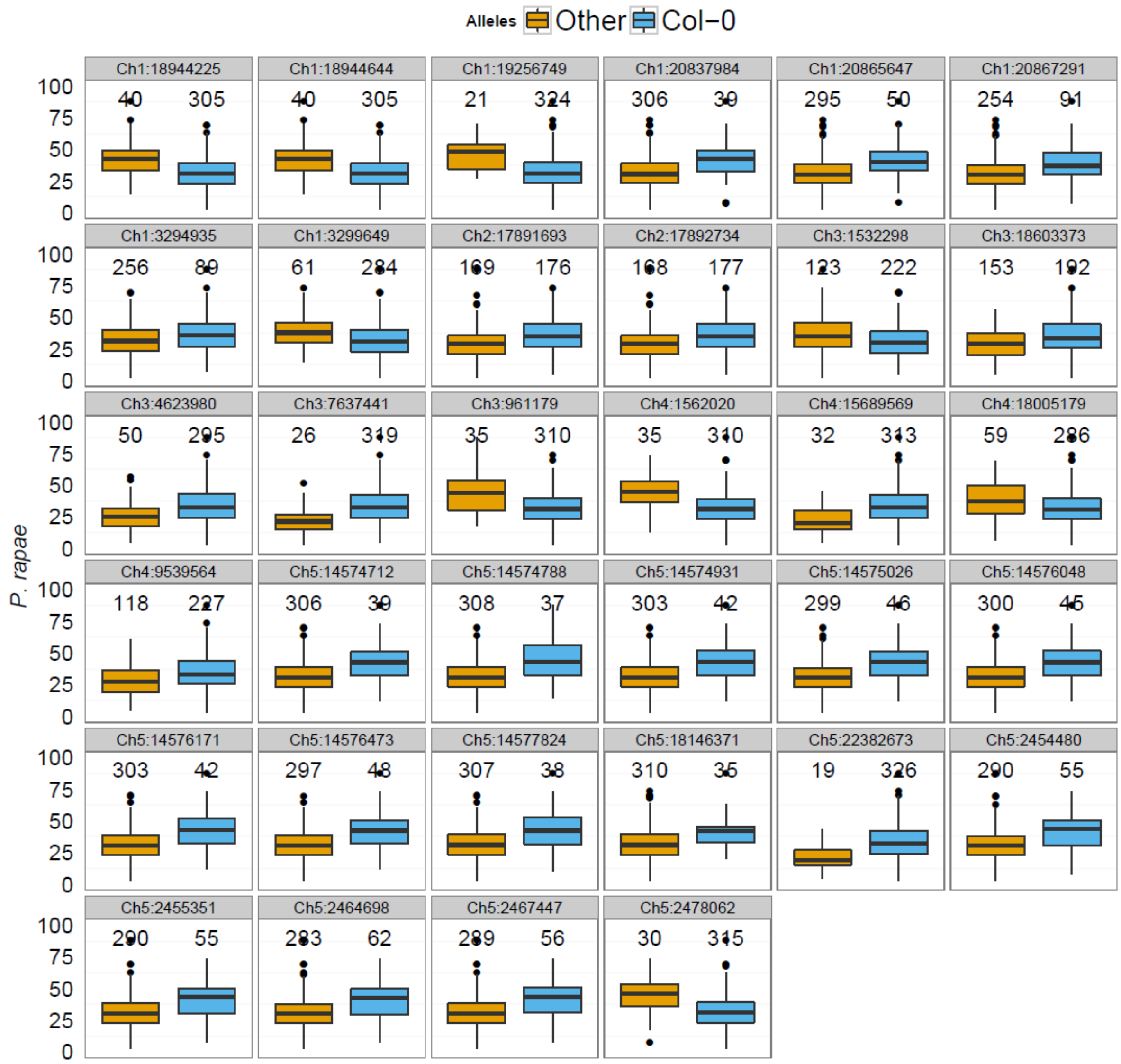

Fig. S3 Allele effects of significant SNPs associated with *Arabidopsis thaliana* rosette fresh weight reduction in response to *P. rapae*. Chromosome and SNP position are indicated. Number of accessions having the Col-0 allele or another allele (Other) are indicated above the boxes. *Y*-axis represents the percentage of biomass reduction compared to the control situation. Box plots represent median value (thick line), the first plus third quartiles (box), and the whiskers represent the lowest and highest values; the circles represent outliers.

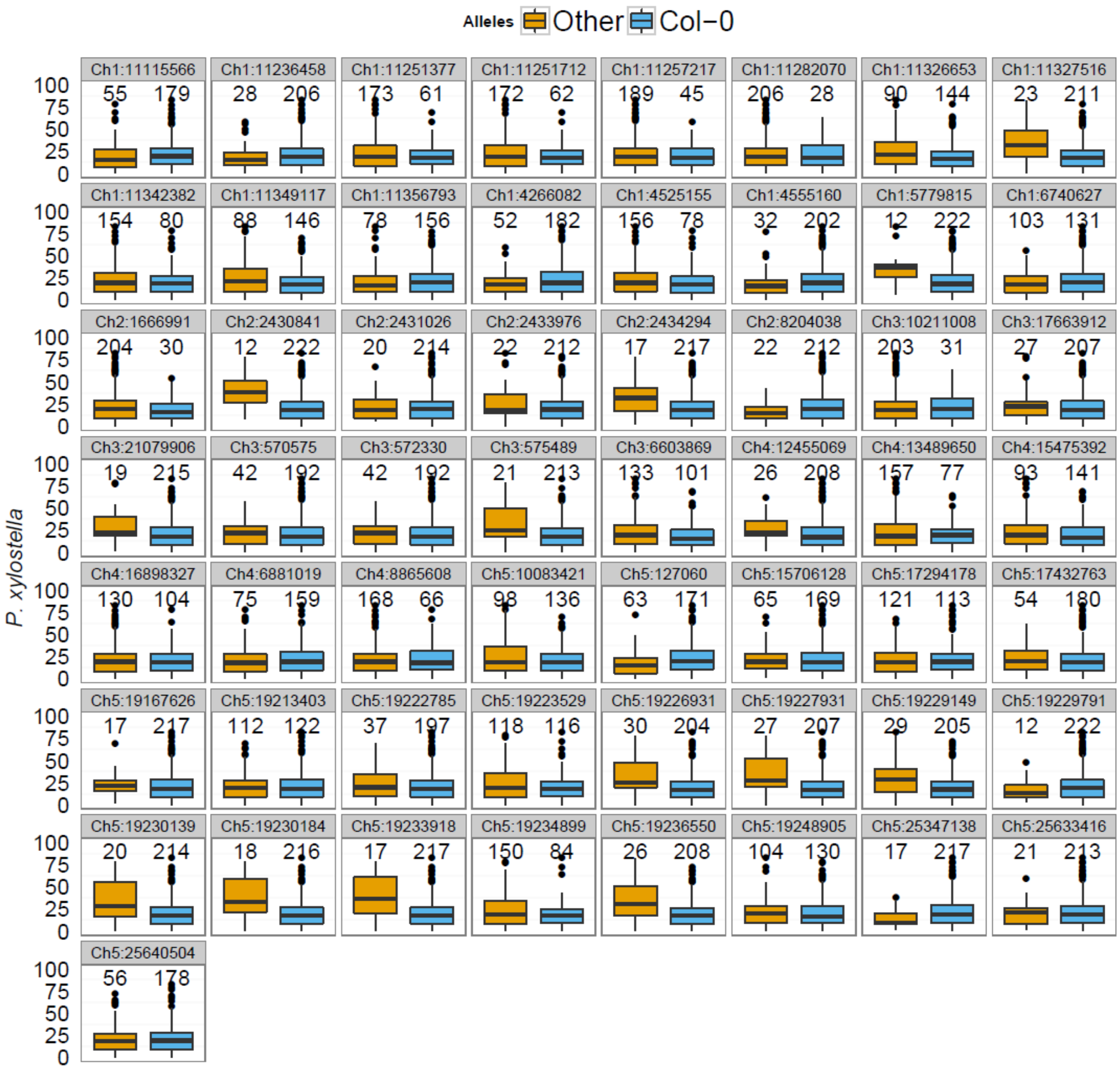

Fig. S4 Allele effects of significant SNPs associated with *Arabidopsis thaliana* rosette fresh weight reduction in response to *P. xylostella*. Chromosome and SNP position are indicated. Number of accessions having the Col-0 allele or another allele (Other) are indicated above the boxes. *Y*-axis represents the percentage of biomass reduction with respect to the control situation. Box plots represent median value (thick line), the first plus third quartiles (box), and the whiskers represent the lowest and highest values; the circles represent outliers.

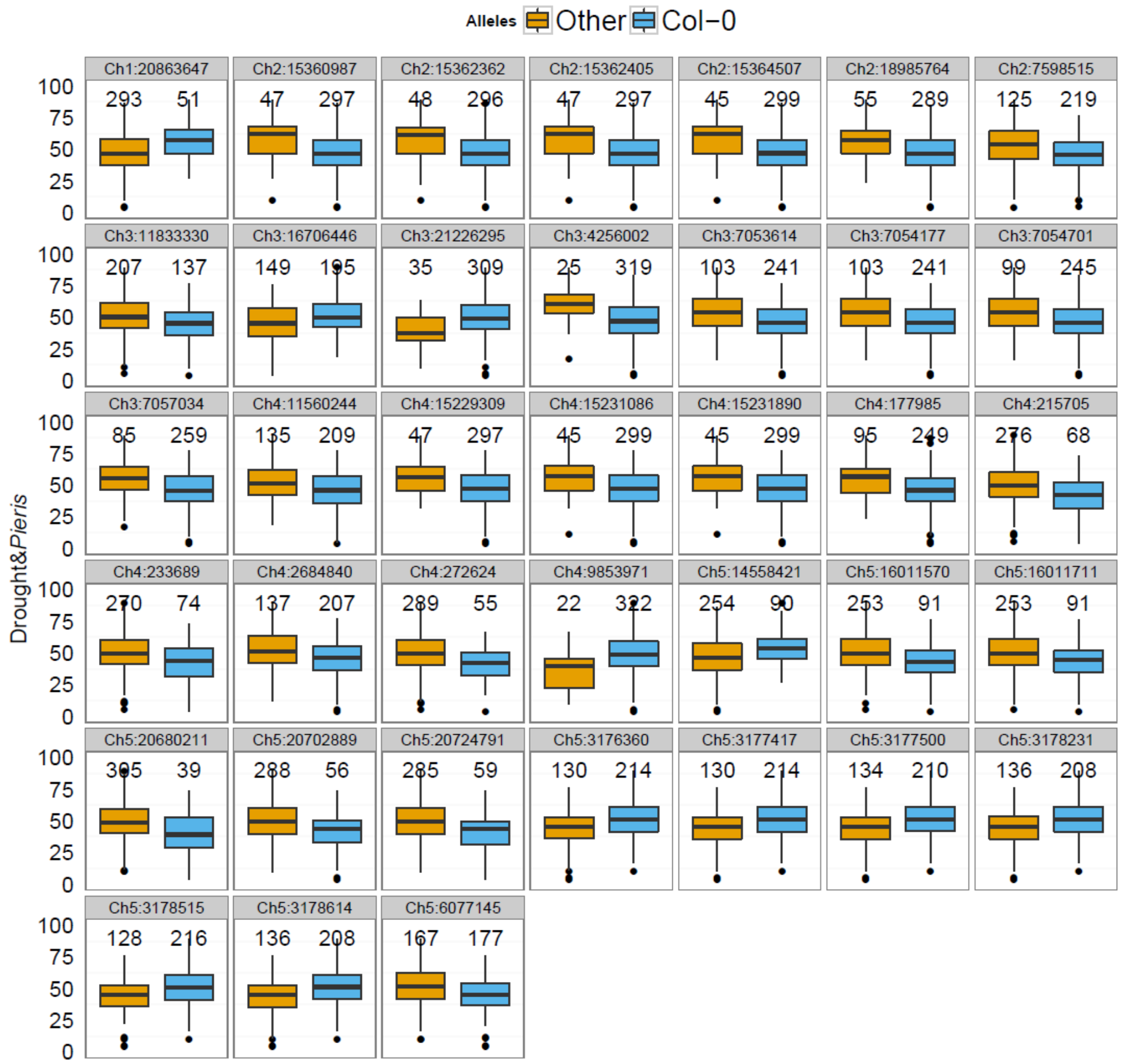

Fig. S5 Allele effects of significant SNPs associated with *Arabidopsis thaliana* rosette fresh weight reduction in response to drought and *P. rapae*. Chromosome and SNP position are indicated. Number of accessions having the Col-0 allele or another allele (Other) are indicated above the boxes. *Y*-axis represents the percentage of biomass reduction with respect to the control situation. Box plots represent median value (thick line), the first plus third quartiles (box), and the whiskers represent the lowest and highest values; the circles represent outliers.

New Phytologist

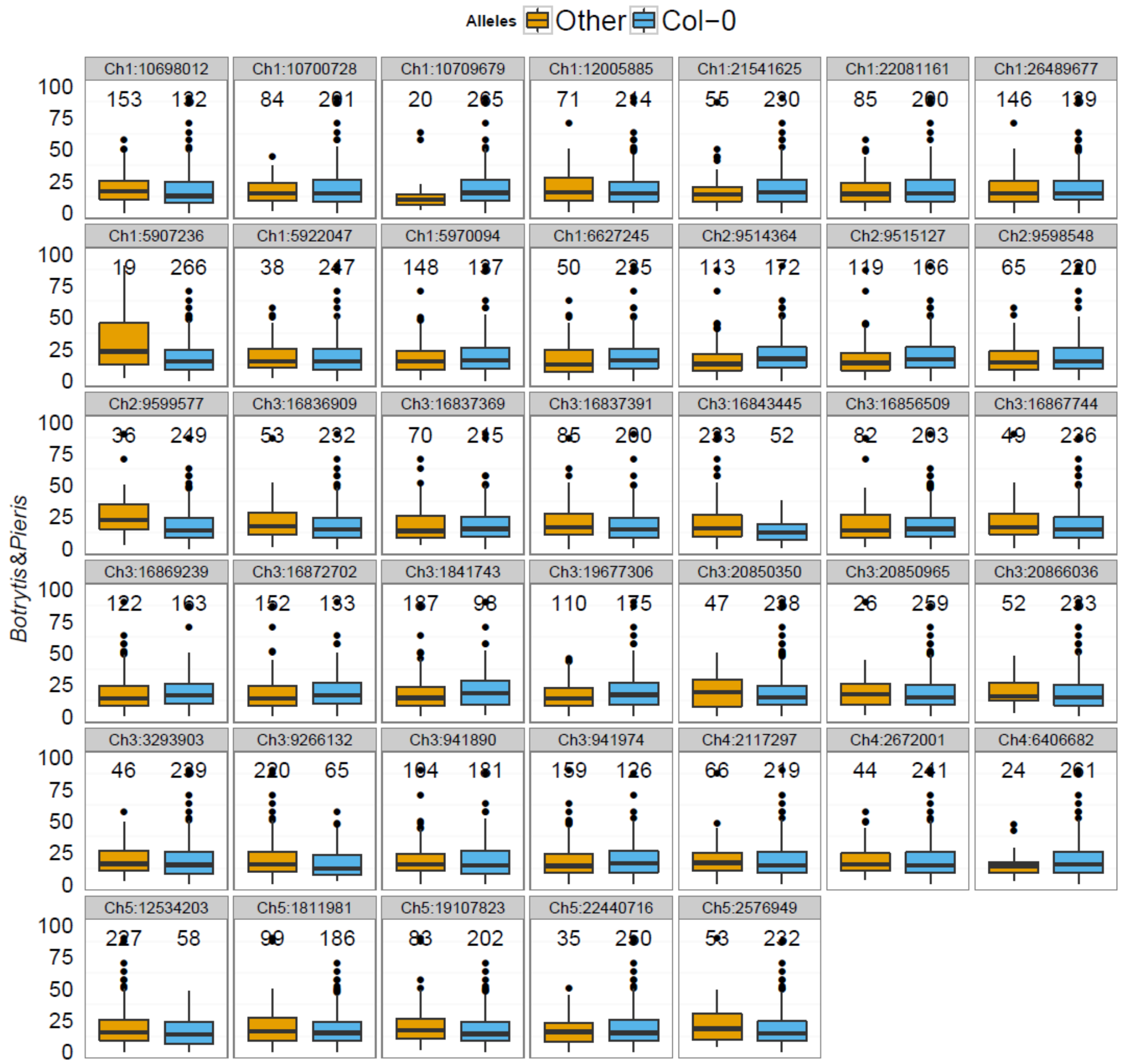

Fig. S6 Allele effects of significant SNPs associated with *Arabidopsis thaliana* rosette fresh weight reduction in response to *B. cinerea* and *P. rapae*. Chromosome and SNP position are indicated. Number of accessions having the Col-0 allele or another allele (Other) are indicated above the boxes. *Y*-axis represents the percentage of biomass reduction with respect to the control situation. Box plots represent median value (thick line), the first plus third quartiles (box), and the whiskers represent the lowest and highest values; the circles represent outliers.
